# Diagnostic et prise en charge d´une thrombopénie néonatale sévère par allo-immunisation materno-fœtale: rapport d´un cas et revue de la littérature

**DOI:** 10.11604/pamj.2020.37.382.24325

**Published:** 2020-12-29

**Authors:** Jihane Toughza, Aomar Agadr, Mouad Nejjari, Insaf Al Ammari

**Affiliations:** 1 Department of Pediatrics, Mohammed VI University of Health Sciences, Casablanca, Morocco,; 2 Department of Pediatrics, Mohammed V, Rabat, Morocco

**Keywords:** Thrombopénie néonatale sévère, allo-immunisation materno-fœtale, diagnostic, prise en charge, Severe neonatal thrombocytopenia, neonatal thrombocytopenia due to maternal alloimmunization, diagnosis, management

## Abstract

La thrombopénie est une anomalie hématologique fréquente durant la période néonatale particulièrement chez les nouveau-nés hospitalisés en unité de soins intensifs et chez les prématurés. Elle est définie par une numération plaquettaire inférieure à 150 000/mm^3^. Parmi les thrombopénies immunes, la thrombopénie par allo-immunisation materno-fœtale d´incidence de 1 cas pour 1 000 naissances, n´est pas un évènement rare mais souvent non diagnostiqué dans les formes mineures. Celle-ci résulte de l'immunisation maternelle contre les antigènes plaquettaires fœtaux hérités du père et absents chez la mère. Les allo anticorps IgG maternels traversent le placenta et provoquent la destruction des plaquettes fœtales. En cas de thrombopénie sévère, les conséquences sont graves avec 10 à 30% d´hémorragies intracrâniennes. Le diagnostic est essentiellement clinique, et doit être connu par les pédiatres.

## Abstract (en)

Thrombocytopenia is a common hematologic disorder in the neonatal period. It can occur in neonates hospitalized in the Intensive Care Unit and in preterm infants. It is characterized by a platelet count of less than 150.000/mm^3^. In the context of immune thrombocytopenia, neonatal thrombocytopenia due to maternal alloimmunization (estimated at 1 per 1000 live births) is not a rare event but it is often undiagnosed in minor forms. This is caused by maternal immunization against fetal platelet antigens inherited from the father and lacking in the mother. Maternal IgG alloantibodies cross the placenta causing fetal platelet destruction. In severe thrombocytopenia, consequences can be severe, with intracranial bleeding occurring in 10-30% of patients. Diagnosis is essentially based on clinical examination and must be suspected by pediatricians.

## Introduction

Dans le cas de la thrombopénie par l'incompatibilité plaquettaire materno-fœtale, les anticorps maternels IgG traversent le placenta et reconnaissent pour cibles les plaquettes fœtales. La thrombopénie qui en résulte peut être très sévère (inférieure à 20.10^9^/L) et à l'origine d'hémorragie intracérébrale (HIC) in utero ou lors de l'accouchement. Les conséquences en sont graves avec dans 25% des cas mort fœtale ou séquelles neurologiques [1]. Dans environ la moitié des cas la thrombopénie fœtale liée à l'antigène HPA-1a surviennent dès la première grossesse et le risque de récidive est de plus de 80% lors d'une grossesse ultérieure avec fœtus incompatible [2]. En effet, dans la population caucasoïde l'antigène plaquettaire (Human Platelet Antigen) HPA-1a est le plus souvent impliqué. Nous présentons une observation d´une thrombopénie sévère néonatale associée à un hématome cervical chez un nouveau-né, dans un contexte d´une mère seconde pare conduisant au diagnostic d'allo-immunisation materno-fœtale. Les possibilités de prise en charge des grossesses suivantes sont également discutées.

## Patient et observation

Bébé X est un nouveau-né de sexe masculin. Il est né au terme de 39 semaines de gestation, après une grossesse sans particularité, et un accouchement par voie basse (extraction instrumentale). Le poids de naissance était de 3,5 kilogrammes, le score d´Apgar à 10/10. La mère, Mme Z., âgée de 30 ans, II gestes, I pare, de groupe sanguin O Rhésus positif, avait pour principal antécédent, un avortement spontané précoce à 10 semaines de gestation, un an auparavant.

A la naissance il présentait une petite bosse sérosanguine droite. En 24 heures d´évolution, sont apparus, un hématome cervical droit, un purpura pétéchial diffus, et des ecchymoses aux membres inférieurs. Le bilan biologique avait confirmé la thrombopénie à 4 000 G/mm^3^ associée à une anémie à 6,6 g/dl normocytaire, régénérative (réticulocytes à 127 G/mm^3^), hémolytique à bilirubine libre (bilirubine totale 145 mg/l bilirubine libre 140 mg/l bilirubine conjuguée 5 mg/l, LDH 437UI/l, haptoglobine 0,33 g/l). Le test de Coombs était négatif. Le groupe sanguin était 0 Rhésus positif. L´échographie transfontanellaire (ETF) mettait en évidence une hémorragie intraventriculaire de grade II. La numération formule sanguine de la mère était sans particularité. Toutes les sérologies infectieuses étaient revenues négatives, ainsi que les anticorps anti nucléaires, et les antiDNA natifs. Le myélogramme était en faveur d´une moelle riche, ou toutes les lignées étaient représentées, avec des mégacaryocytes à tous les stades de maturation.

Bébé X a bénéficié d´une transfusion en culot globulaire érythrocytaire ayant permis la correction de l´hémoglobine (hémoglobine de contrôle à 15,6 g/dl; bilirubine totale 80 mg/l; bilirubine libre 72 mg/l; bilirubine conjuguée 8,6 mg/l). La thrombopénie est restée réfractaire (inférieure à 50G/mm^3^) aux transfusions itératives d´unités plaquettaires non phénoypées, ainsi qu´aux perfusions d´immunoglobulines intraveineuses (IgG IV) associées à la corticothérapie. Bébé X a finalement bénéficié d´une transfusion de plaquettes maternelles irradiées à J12 de vie, associée à une corticothérapie, ayant permis la correction de la numération plaquettaire à 103 G/mm^3^) puis à 318 G/mm^3^. Un bilan d'immunologie plaquettaire a été réalisé, mettant en évidence une allo-immunisation materno-fœtale dans le système HPA-1, puisque la mère était HPA-1b homozygote et le père HPA-1a homozygote, et qu'il existait un allo-anticorps anti HPA-1a dans le sérum maternel. Le bilan a également révélé la présence d´autoanticorps antiplaquettes circulants et fixés, participant très probablement à la bicytopénie initiale.

L´évolution ultérieure fut favorable, à partir de J11 de traitement, un sevrage progressif de la corticothérapie fut débuté. L´ETF de contrôle était sans particularité. Un dosage des auto-anticorps anti-plaquettes chez la mère a été réalisé à trois mois. La nécessité d´une surveillance pluridisciplinaire en milieu spécialisé a été expliquée à la mère pour les grossesses ultérieures. Une quantification initiale puis un suivi des anticorps anti HPA-1a chez la mère furent programmés. La mère bénéficiera d´une perfusion d´immunoglobulines à partir de 18 semaines d´aménorrhées dont le rythme dépendra des taux d´anticorps.

## Discussion

La thrombopénie par allo-immunisation fœtale ou néonatale (TAIFN) est confirmée par la mise en évidence d´un allo-anticorps maternel dirigé contre un antigène paternel présent chez l´enfant et absent chez la mère [3]. Un nouveau-né sur 1 000 est probablement atteint dans le cas de l'allo-immunisation anti HPA-1a. En tenant compte des autres systèmes allo-antigéniques (essentiellement HPA-3 et 5 dans la population caucasoïde) la fréquence de l'atteinte serait en moyenne de 1,5/1 000 enfants vivants [2, 4]. Il n´existe pas de signe prédictif d´atteinte fœtale ou néonatale, les nouveaux nés peuvent être asymptomatiques ou présenter un tableau clinique avec des pétéchies, des ecchymoses et une hémorragie intracrânienne (HIC) [5]. Ces thrombopénies se compliquent dans 10 à 30% des cas d´HIC [6]. Cinquante-quatre pourcent (54%) des HIC se produisent avant 28 semaines de gestation, entrainant des troubles neurologiques graves chez 83% des survivants, avec une évolution fatale dans 35% des cas [7].

Le taux d'anticorps maternels ne permet pas de prédire le degré de la thrombopénie fœtale, en effet dans 15 à 20% des cas, aucun anticorps n'est mis en évidence malgré l'utilisation de tests spécifiques sensibles (MAIPA-Test) [8, 9]. Il n'y a pas d'amélioration spontanée de la thrombopénie au cours de la grossesse et lorsque des numérations plaquettaires fœtales sont répétées on constate une aggravation [10]. En général, le risque de récidive est supérieur à 80% avec une atteinte au moins aussi sévère pour tout fœtus incompatible [8]. L'échographie transfontanellaire est urgente devant la suspicion de TAIFN pour éliminer tout saignement. L'imagerie par résonance magnétique peut être réalisée si nécessaire pour confirmer ou identifier un HIC [11]. La numération plaquettaire typique du nadir chez le patient porteur d´une TAIFN se produit dans les 48h de vie [12]. Le but du traitement des TAIFN est de prévenir et de minimiser les hémorragies majeures, telles que l´HIC ou le saignement gastro-intestinal, pouvant être fatales pour le patient [13].

La thérapeutique initiale est, suivant les protocoles, fonction du risque évalué, puis adaptée en fonction de la réponse au traitement: transfusion plaquettaire, association des corticostéroïdes aux IgGIV, augmentation des doses d'IgGIV (2 g/kg) et des corticostéroïdes (2 mg/kg/j). Les transfusions plaquettaires restent le traitement de première intention des TAIFN, et notamment les transfusions de plaquettes sélectionnées par HPA (maternelles ou donneuses). En effet, ces dernières semblent apporter un double bénéfice par rapport aux plaquettes non sélectionnées par HPA, sur l´efficacité et la durée de réponse [14]. L´étude réalisée par Mueller-Eckhardt *et al*. [15], a montré que le nombre de plaquettes est passé à plus de 50 G/mm^3^chez 83% des nouveau-nés ayant reçu des plaquettes maternelles versus 38% qui ont été transfusés avec des plaquettes non sélectionnées par HPA. La demi-vie des plaquettes sélectionnées HPA-1a / 5b transfusées était estimée à deux fois celle des plaquettes non sélectionnées [15].

Des recommandations ont été établies. En effet, la prise en charge thérapeutique de la thrombopénie néonatale dans les 24 premières heures de vie dépendra de la numération plaquettaire (NP) [1, 16]. Le nouveau-né bénéficiera d´une transfusion prophylactique si la NP est inférieure à 30 G/mm^3^, à partir d´un donneur HPA compatible ou de la mère si cela est réalisable [17-20]. En cas d´indisponibilité de produit compatible, des plaquettes non phénotypées sont associées à un traitement par immunoglobulines IV.

Lorsque la NP montre une thrombopénie supérieure à 30 G/mm^3^, le nouveau-né sera surveillé sur le plan clinique jusqu´à l´obtention d´un taux plaquettaire = 100 G/mm^3^. Bien que les transfusions conduisent à des augmentations du nombre de plaquettes chez le nouveau-né, les études nécessitent des échantillons plus importants pour confirmer si les transfusions de plaquettes réduisent la morbidité et / ou la mortalité néonatales (1), ainsi que pour évaluer le bénéfice de l´ajout des corticoïdes aux transfusions de plaquettes et / ou aux perfusions d´IgG IV chez les nouveau-nés [12, 21, 22]. La majorité des nourrissons évoluent favorablement dans un délai de 1 à 5 semaines [23]. Auquel cas, des diagnostics alternatifs doivent être explorés.

La récurrence de l'atteinte souvent plus sévère lors des grossesses successives et l'absence de paramètres maternels prédictifs de l'atteinte fœtale justifient une prise en charge spécifique de la grossesse [24-27]. Une surveillance échographique rigoureuse devra être instituée dès le début du second trimestre de la grossesse [28]. La ponction de sang fœtal (PSF), seul examen permettant d'évaluer la thrombopénie, doit être réservée à des équipes spécifiquement entraînées. Le risque du geste lui-même est majoré dans ce contexte et une transfusion plaquettaire extemporanée doit pouvoir être réalisée pour certains lors de toute PSF surtout en cas de thrombopénie profonde [8]. Deux prélèvements fœtaux semblent au minimum nécessaires à 20-22 SA et en fin de grossesse. Les transfusions plaquettaires in utero répétées associées à une extraction fœtale dès la maturité pulmonaire atteinte sont à réserver aux formes les plus sévères.

## Conclusion

La prise en charge diagnostique et thérapeutique de la thrombopénie néonatale par allo-immunisation plaquettaire materno-foetale est une urgence vitale. Sa recherche doit être systématique devant toute thrombopénie néonatale. La transfusion de plaquettes maternelles ou d´un donneur HPA compatible reste la thérapeutique apportant le meilleur bénéfice. L´hémorragie intracérébrale est la complication la plus à craindre. Une prise en charge spécifique en milieu spécialisé lors des grossesses ultérieures est à expliquer puis à programmer pour les mamans.
